# Synthesis and Biomedical Applications of PLA-HPG-Based Biodegradable Nanocarriers: A Review

**DOI:** 10.3390/bios15080502

**Published:** 2025-08-03

**Authors:** Yijun Shen, Xuehan He, Lei Chen

**Affiliations:** 1 Guangdong Provincial Key Laboratory of Sensor Technology and Biomedical Instrument, School of Biomedical Engineering, Sun Yat-sen University, Shenzhen 518107, China; 2 School of Pharmaceutical Sciences (Shenzhen Campus), Sun Yat-sen University, Shenzhen 518107, China

**Keywords:** PLA-HPG, biodegradable nanocarriers, drug delivery, wound dressing, sunblock

## Abstract

The development of biodegradable nanocarriers has long been a priority for researchers and medical professionals in the realm of drug delivery. Because of their inherent benefits, which include superior biocompatibility, customizable degradability, easy surface functionalization, and stealth-like behavior, polylactic acid-hyperbranched polyglycerol (PLA-HPG) copolymers have demonstrated a promising future in the field of biomedical research. The synthesis of PLA-HPG copolymers and the creation of their nanoparticles for biomedical uses have been the focus of current efforts. In this review, we summarize the synthetic strategies of PLA-HPG copolymers and corresponding nanoparticles, and highlight their physicochemical properties, biocompatibility, and degradation properties. Furthermore, we introduce a number of PLA-HPG nanoparticles that are utilized for surface skin delivery, wound dressing, and in vivo drug delivery biological applications. Finally, we conclude by offering our thoughts on how this nanoplatform might advance in the future.

## 1. Introduction

In the field of biomedicine, biodegradable nanocarriers have become a key strategy to improve therapeutic efficacy while minimizing the side effects of bioactive molecules [1]. Among polymeric systems, polyglycerol (PG)-based polymers have garnered particular interest due to their outstanding biocompatibility, high water solubility, antifouling properties, and easy-tunable chemical functionalization [2]. Nevertheless, classic linear PG, despite its hydrophilic nature and biological innocuity, has limited effectiveness in the surface modification of nanoparticles (NPs) [3].

To address these limitations, hyperbranched polyglycerol (HPG), a highly branched and globular structural derivative of PG, has been developed as an advanced alternative. Its three-dimensional structure increases the density of peripheral hydroxyl groups, facilitating enhanced chemical modification and multivalent conjugation. Additionally, its dendritic architecture enhances antifouling properties and protein resistance compared to linear PG [4]. For instance, surfaces coated with HPG—especially when applied using grafting-from techniques—form compact, hydrated polymer layers that effectively resist nonspecific protein adsorption [5].

In parallel, polyethylene glycol (PEG), being the most commonly applied hydrophilic polymer for modifying nanoparticle (NP) surfaces, also exhibits excellent antifouling and biocompatibility. It has been extensively utilized in drug delivery to prolong circulation time [6] and minimize immunogenic responses [7]. However, PEG has drawbacks, including poor oxidative stability, non-biodegradability, and limited modification sites due to its linear structure [8]. HPG outperforms PEG by maintaining desirable stealth characteristics while offering enhanced structural stability and more accessible functional groups, thanks to its hyperbranched structure with a higher density of hydroxyl groups on its surface. This structural advantage makes HPG more resistant to protein adsorption than PEG [9].

Moreover, HPG outperforms linear PG and PEG in terms of pharmacokinetics and biosafety. In vivo studies have shown that HPG has a longer blood circulation half-life and lower renal clearance, attributed to its compact globular form, which is less prone to rapid glomerular filtration [10]. Its superior hemocompatibility and minimal complement activation have also been demonstrated, even with high systemic doses [10]. Collectively, these advantages position HPG as a highly valuable alternative to linear PG and PEG as a hydrophilic component in the development of biodegradable nanocarriers, enabling its successful application in various biomedical fields, including tumor-targeted drug delivery [11], gene transfer [12], and tissue engineering [13].

While HPG offers several benefits, its non-biodegradability limits its use in biomedical applications, particularly in prolonged scenarios involving drug delivery where complete biodegradation is necessary [14]. Polylactic acid (PLA) is a widely studied aliphatic polyester recognized for its outstanding biodegradability. It is hydrophobic and works well with hydrophobic drugs, enzymes, and proteins [15]. Thus, PLA is now a very promising biopolymer in biomedical applications such as drug delivery [16], gene transfer [17], surgical sutures [18], tissue engineering [19], and regenerative medicine [20]. Among synthetic polymers, PLA is the most commonly used material for electrospinning and is frequently employed as a copolymer or in conjunction with other biocompatible polymers such as polyglycolic acid (PGA) and poly(ε-caprolactone) (PCL), along with their respective copolymers [21]. By grafting PLA onto a hydroxypropyl guar (HPG) core or blending the two polymers, amphiphilic structures can be created that leverage PLA′s biodegradability and drug-encapsulating abilities, along with the hydrophilic surface properties of HPG [22].

**Figure 1:**
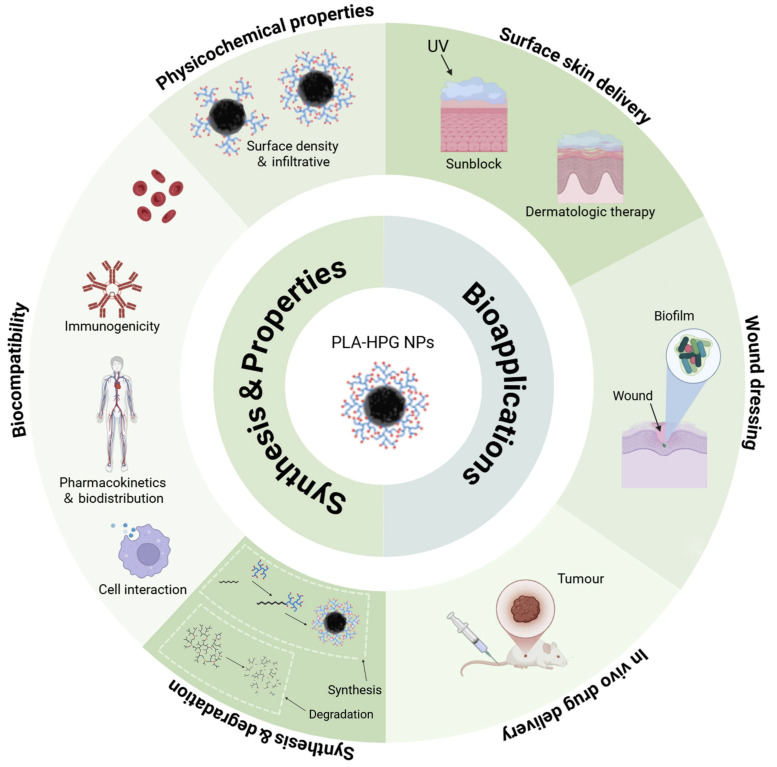
The illustration of the content of this review includes synthesis, properties, and bioapplications of PLA-HPG NPs as nanocarriers. Reproduced with permission from [22,23]. Copyright 2022 Elsevier Ltd. and 2012 American Chemical Society. All rights reserved.

This review provides a summary of the most recent synthesis methods for PLA-HPG nanocarriers and their uses in the biomedical field, particularly concerning skin surface delivery, wound dressing, and in vivo drug delivery (Figure 1).

## 2. Synthesis of PLA-HPG

### 2.1. Synthesis of HPG

**Figure 2:**
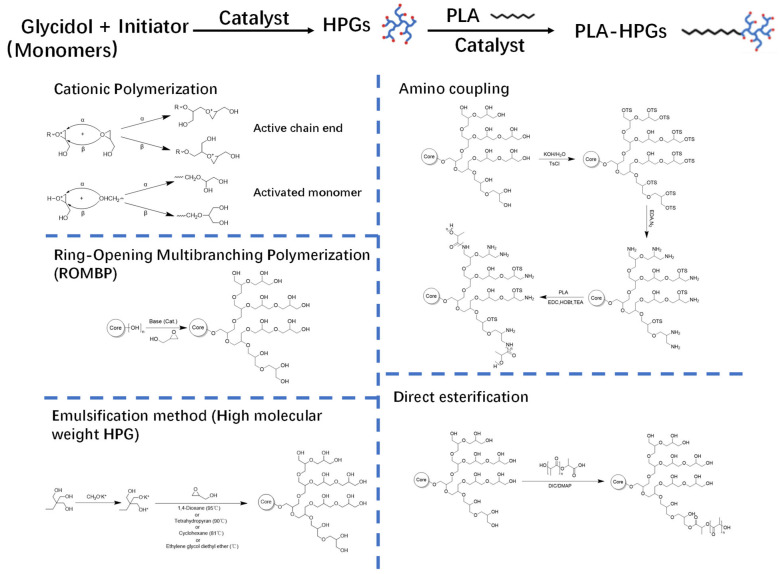
Schematic illustration of the synthetic methods of HPGs (**left**) and PLA-HPGs (**right**). Reproduced with permission from [22]. Copyright 2022 Elsevier Ltd. All rights reserved.

Table 1 summarizes the synthesis and properties of HPG and PLA-HPG polymers. The synthesis of HPG serves as the foundation for preparing PLA-HPG (Figure 2-left). Following Flory’s AB2 model of monomer distribution [24], Sandler and Berg first synthesized HPG through the ring-opening polymerization of glycidol at room temperature, utilizing simple bases such as sodium methoxide and potassium hydroxide [25]. This reaction produced highly branched, low-molecular-weight polymers (Mn ≈ 445 g/mol) with excellent water solubility. However, the reaction was exothermic and challenging to manage, resulting in broad molecular weight distributions and poor reproducibility.

The low molecular weights and structural imprecision limited their use in areas requiring precise size and structure.

Dworak et al. reported a cationic polymerization technique utilizing Brønsted and Lewis acids to achieve improved control over polymer structure [26]. This method produced polymers with moderate molecular weights (2.5~6.0 kDa) and more adjustable branching. Structural characterization demonstrated the ability to vary the ratio of linear-to-branching units by modifying the catalyst or the reaction temperature. Although this approach represented an advancement over base catalysis, it still could not achieve high molecular weights and involved acidic conditions that could complicate biomedical applications.

Frey’s group developed anionic ring-opening multibranching polymerization (ROMBP), initiated with trimethylolpropane (TMP) and using potassium methoxide as the base [27], with greater control over the molecular weight (up to ~6.0 kDa) and polydispersity index (PDI < 1.3) resulting in well-defined, amphiphilic hyperbranched polyglycerols (HPGs) suitable for forming unimolecular micelles. Although the structural uniformity and functionalizability improved compared to earlier methods, the achievable molecular weight ranges remained somewhat limited.

To overcome the limitation in molecular weight, Kizhakkedathu et al. developed a solvent-assisted ROMBP approach [28]. Polar solvents, such as 1,4-dioxane with enhanced cation solvation and alkoxide nucleophilicity, were introduced into the system to enable the synthesis of HPGs with molecular weights of up to 500 kDa and a low PDI of approximately 1.05. This solvent system also promoted uniform growth and prevented premature termination of the reaction. The resulting high-quality, size-controlled HPGs exhibited excellent water solubility and hydroxyl accessibility, making them ideal for biomedical applications that require well-defined hydrodynamic properties and consistent surface functionality.

### 2.2. Synthesis of PLA-HPG Copolymers

Conventional ring-opening polymerization of cations and anions to prepare HPG is based on toxic initiators, catalysts, or organic solvents, which limits its biomedical applications [29]. Consequently, there have been successful efforts to synthesize HPG using biocompatible initiators and water-based solutions. In 2018, Mohsen Adeli et al. discovered that ascorbic acid can serve as an initiator for the ring-opening polymerization of glycidol, resulting in the formation of highly biocompatible and biodegradable HPG with a degree of branching (DB) ranging from 0.44 to 0.53 [30]. The PDI is a function of temperature and the molar ratio of glycidol to ascorbic acid (G/As). At a constant temperature, an increase in the G/As ratio leads to a corresponding increase in the PDI. Since HPG synthesized through this method does not contain toxic residues, it is particularly well suited for biomedical applications.2.2. Synthesis of PLA-HPG Copolymers

The synthesis of PLA-HPG copolymers optimizes their properties and applications. Direct esterification and amino coupling are the two primary methods used in the synthesis of PLA-HPG copolymers (Figure 2–right).

In the amino coupling method, HPG is first aminated by converting a portion of its numerous hydroxyl groups into primary amine groups through a separate synthetic step. These amine-terminated HPGs are then coupled with carboxyl-terminated PLA chains via an amide-forming process mediated by carbodiimide [31]. The amino coupling technique can achieve high coupling efficiency; however, any unused amine groups remaining on HPG after coupling may impart a positive charge, reducing its ′stealth′ hydrophilicity and biocompatibility as an HPG corona [32].

The direct esterification method forms ester bonds between PLA and HPG in a single step, without the need for prior modification of their respective functionalities, thereby preserving HPG’s native polyol characteristics [4]. An application of this method involves the direct condensation of the hydroxyl groups of HPG with the carboxyl end-groups of PLA, utilizing dehydration coupling reagents or catalysts (e.g., carbodiimides such as DCC in conjunction with DMAP as a catalyst) to facilitate the formation of ester bonds [4]. This one-pot reaction effectively grafts PLA chains onto HPG through ester bonds, eliminating the need for intermediate amine derivatization.

### 2.3. Fabrication of PLA-HPG NPs

The nanoprecipitation method is commonly employed for the encapsulation of hydrophilic and hydrophobic molecules [33]. For PLA-HPG nanocarriers, the copolymer is usually dissolved in a small amount of DMF or acetonitrile and occasionally mixed with methanol for better solubilization. The solution is added drop by drop to an excess aqueous phase under agitation. The amphiphilic copolymer spontaneously assembles into NPs because of the PLA segment’s hydrophobic interaction and is held together by HPG chains on the outer surface. Later, dialysis or ultrafiltration is commonly used to extract excess solvent and unencapsulated agents [22].

Nanoprecipitation is advantageous owing to its simplicity and scalability, as well as its avoidance of high-energy input; but the technique is restricted to hydrophobic or amphiphilic drugs, and solubilization is dependent upon copolymer solubilization in chosen solvents [34]. Thus, the poor encapsulation efficacy of hydrophilic drugs could be a disadvantage unless additives are added to adjust hydrophilic core conditions.

## 3. Properties of PLA-HPG

### 3.1. Physicochemical Properties

PLA-HPG copolymers spontaneously form core-shell NPs with the desired size and stability in aqueous solution. Micelle formation is driven by the PLA segments and is steric and hydrated by the HPG shell. Gao et al. reported that PLA-HPG NPs have a typical spherical morphology, with diameters ranging from ~66 to 182 nm, depending on the PLA molecular weight and HPG ratio [32]. Higher molecular weights of PLA lead to larger core structures, thus influencing the overall NP size. For example, NPs formed with PLA of molecular weight 21 kDa exhibit an average size of approximately 115 nm, while those made with PLA of 60 kDa have a size of approximately 182 nm. This occurs because longer PLA chains result in increased hydrophobic interactions, which further influence the aggregation behavior and size of the particles. This is particularly advantageous for encapsulating hydrophobic drugs like camptothecin (CPT), where higher-molecular-weight PLA improves the drug loading capacity and retention within the NP core [9,32].

On the other hand, increasing the proportion of HPG in the copolymer leads to the improved dispersion and enhanced stability of the NPs. When the HPG ratio increases from 9.4% to 33.3%, the water sorption capacity of the NPs increases significantly, which helps maintain their size stability in aqueous environments. A higher HPG grafting density also contributes to increased resistance to nonspecific protein adsorption, which is critical for reducing rapid clearance by the mononuclear phagocyte system. For instance, NPs with a dense HPG corona exhibit extended blood circulation and decreased liver accumulation compared to PEG-coated analogs [12,22].

These compositional variations also affect the drug encapsulation efficiency. Studies by Gao et al. demonstrated that PLA21-HPG NPs achieved BSA loading capacities up to 23%, with an encapsulation efficiency of approximately 86%, highlighting the importance of tuning the copolymer composition for optimal drug delivery outcomes [11]. The physicochemical properties of PLA-HPG NPs are sensitive to their composition. Additional studies have established the influence of copolymer structure on protein encapsulation and aqueous properties. Gao et al. verified that a higher level of HPG results in significantly enhanced water sorption and improved NP dispersion [31]. The dense branching of the HPG structure leads to reduced surface energy and, thus, better aqueous compatibility than in conventional PLA systems. The surface zeta potential changes from weakly positive to negative values upon the encapsulation of proteins such as bovine serum albumin (BSA), indicating partial adsorption at the particle interface. This observation is confirmed by X-ray photoelectron spectroscopy (XPS) data, which reveals surface-exposed sulfur derived from protein molecules.

PLA-HPG formulations have been directly compared to PLA-PEG analogs and have been found to exhibit similar size and encapsulation capacities but with superior colloidal properties under biological conditions. The branched structure of HPG facilitates the formation of more compact and hydrated surface layers, resulting in significantly reduced NP aggregation over time and enhanced suspension stability. For instance, Gao et al. reported that PLA-HPG NPs with a PLA molecular weight of 21 kDa and HPG content of 25% exhibited an average diameter of ~115 nm and a BSA encapsulation efficiency of 86%, comparable to PEG-PLA NPs of similar composition but with better aqueous dispersibility and reduced aggregation over 7 days [31].

In terms of bioadhesion, Mai et al. demonstrated that bioadhesive PLA-HPG NPs exhibited a 4-fold increase in retention time on psoriatic skin lesions compared to PEGylated controls after washing tests, suggesting improved topical residence [35].

Regarding systemic circulation, Gao et al. found that PLA-HPG NPs exhibited a plasma half-life of 8.3 ± 1.2 h in mice, which is longer than the 5.1 ± 0.9 h observed for PEG-PLA systems, while simultaneously inducing lower levels of anti-polymer antibodies after repeated administration, indicating lower immunogenicity and better in vivo tolerability [32].

These findings collectively highlight that PLA-HPG nanocarriers not only match PEG-PLA systems in drug encapsulation but surpass them in long-term dispersion stability, bioadhesive strength, and pharmacokinetics, thus offering a compelling alternative for both systemic and localized drug delivery platforms.

### 3.2. Biocompatibility

PLA-HPG NPs demonstrated superior biocompatibility in various tests. They did not induce noticeable hemolysis, platelet activation, or disruption of coagulation parameters during hemocompatibility studies. This is attributed to the hydrophilic nature of the HPG outer shell, which minimizes undesirable interactions with blood [22].

PLA-HPG carriers exhibit lower immunogenicity compared to those based on PEG. Specifically, they do not trigger an antibody-mediated clearance (ABC) effect, which is a common issue with PEG-NPs that leads to rapid elimination upon subsequent dosing due to the presence of anti-PEG antibodies [36]. This advantage is attributed to the hyperbranched architecture of HPG, which forms a densely hydrated and sterically hindered surface that limits the binding of anti-PEG IgM antibodies and subsequent complement activation—a key mechanism underlying the ABC phenomenon in PEGylated systems [12,22,36]. Furthermore, the globular structure and multivalent hydroxyl groups of HPG reduce recognition by splenic marginal zone B cells, which are central to T-cell-independent immune responses triggered by PEG [12]. Reports indicate that HPG-coated NPs do not elicit ABC responses or associated antibodies, even after multiple injections [22]. This indicates that the hyperbranched structure of HPGs of HPG creates a ’stealth’ surface that effectively evades immune recognition.

In vivo, PLA-HPG NPs have less liver accumulation and extended circulation time compared to their counterparts with PEG encapsulation [4]. With sufficient HPG coverage, their protein corona contains fewer opsonins [32]. Cellular uptake by macrophages is minimal, and no cytotoxicity is observed in fibroblasts or endothelial cells, indicating their safety for systemic delivery [22].

### 3.3. Degradation Properties

Under physiological conditions, PLA-HPG NPs degrade through the hydrolytic cleavage of ester bonds in the PLA segments [32]. The rate of degradation is influenced by the composition of the copolymer. NPs with shorter PLA segments and higher proportions of HPG absorb more water and degrade at a faster rate [32]. Beyond polymer composition, environmental factors such as pH, enzymatic activity, and local ionic strength also critically affect the degradation kinetics. For instance, acidic microenvironments (e.g., tumor tissues or intracellular endosomes) can accelerate hydrolysis, while the presence of esterases and proteolytic enzymes enhances polymer breakdown [9,10]. Additionally, smaller particle sizes and higher surface areas facilitate water penetration, further modulating degradation rates. Studies on the release of BSA model protein demonstrate sustained release profiles that are regulated by both diffusion and the relaxation of the polymer matrix [31].

## 4. Biomedical Applications of PLA-HPG-Based Nanocarriers

**Figure 3:**
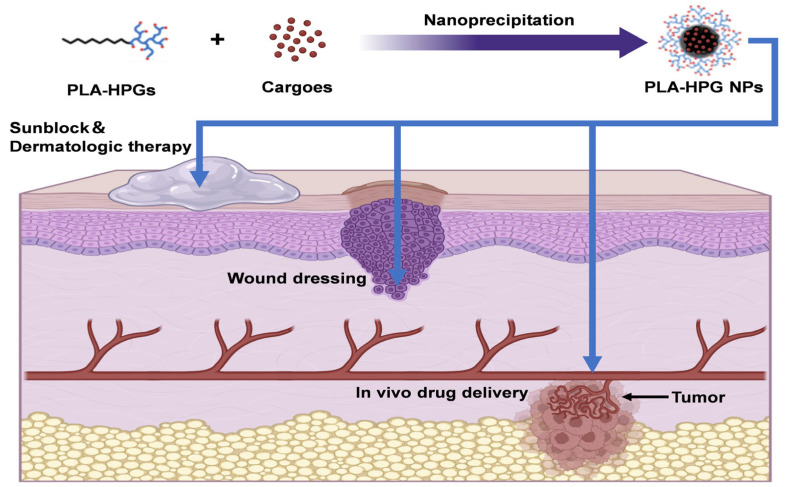
Schematic illustration of the formulation of PLA-HPG NPs and their biomedical applications. Reproduced with permission from [22]. Copyright 2022 Elsevier Ltd. All rights reserved.

As discussed above, PLA-HPG NPs have been proven to reveal the following properties: (1) simple synthesis and tunable surface architecture; (2) excellent hemocompatibility and stealth characteristics; and (3) adjustable degradation rates with sustained drug release. Consequently, PLA-HPG-based nanocarriers have been employed for drug delivery to various sites, including the epidermis, wounds, and internal organs. The relevant patents are listed in Table 2. As shown in Figure 3, next we will summarize the recent advances of PLA-HPG NPs used for (1) surface skin delivery for sunblock or dermatologic therapy, (2) wound dressing, and (3) in vivo targeted drug delivery for tumor treatment reported in reference works.

### 4.1. Surface Skin Delivery for Sunblock or Dermatologic Therapy

**Figure 4:**
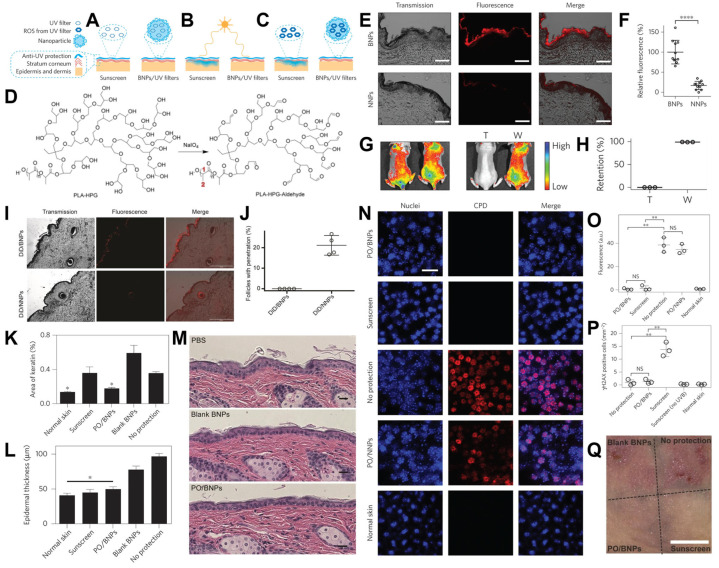
(**A**–**C**) Comparison of BNP-based sunscreen and commercial sunscreen. (**A**) Sunscreen formulations are applied onto the skin. (**B**) After application, commercial sunscreen penetrates into the skin, whereas the BNP formulation remains on the stratum corneum. (**C**) After sunlight exposure, UV filters produce deleterious ROS that can damage adjacent tissue; however, BNPs do not penetrate into the skin and prevent ROS-mediated toxicity by confining these toxic products within the particle. (**D**) Modification of BNPs. (**E**) BNPs and NNPs at 1 mg/mL were incubated on pig skin for six hours in a humidity chamber at 32 °C. (**F**) The fluorescence was quantified and normalized to the average fluorescence of BNPs. (**G**) BNPs encapsulating an infrared dye, IR-780, were applied to the dorsal skin of mice. After wiping with a wet towel (**T**) or washing with water (**W**), their skin retention was imaged with Xenogen. (**H**) The fluorescence after wiping or washing was quantified and normalized to the fluorescence intensity at time zero. (**I**) Fluorescence images showing the penetration of NNPs and BNPs. (**J**) The percentage of follicles penetrated with NPs for DiD-loaded BNPs and NNPs. (**K**,**L**) Epidermal thickness (**K**) and percentage area of keratin (**L**) within the dorsal skin after receiving topical interventions and UV irradiation. (**M**) Evaluation of long-term toxicity of PO/BNPs in vivo. Mice received treatment every other day for six total applications. Afterwards, skin treated with PO/BNPs and blank BNPs was indistinguishable from the PBS control. There was no histologic evidence of cutaneous irritancy, toxicity, or inflammation. (**N**,**O**) CPD staining of mouse dorsal epidermal sheets after receiving different topical interventions and UVB irradiation. (**N**) Epidermal sheets were prepared five minutes after exposure to UVB. (**O**) The fluorescence of CPD on skin receiving different topical interventions was quantified. (**P**) The γH2AXC cells within the epidermis for each intervention were enumerated. (**Q**) Gross skin samples of mouse dorsal epidermis after receiving different topical interventions three days after high-dose UV. Reproduced with permission from [37]. Copyright 2015 Springer Nature Limited.

The development of efficient skin delivery systems has been one of the most significant and long-standing challenges in dermatology. This is particularly evident in the formulation of sunscreens, which must remain within the stratum corneum for prolonged periods to exert protective effects against harmful UV radiation. However, most conventional sunscreen formulations have shown limitations in achieving this goal. At best, they suffer from poor adhesion to the skin surface, resulting in rapid removal by sweat or water. At worst, they penetrate too deeply into the dermis or hair follicles, where they may trigger local irritation, systemic absorption, or even phototoxicity—compromising both efficacy and safety (Figure 4A,C).

To address these issues, PLA-HPG-based nanocarriers have emerged as promising candidates due to their high degree of surface functional versatility, enabling fine-tuned engineering for specific biomedical and dermatological applications [37]. A particularly innovative modification involves the generation of bioadhesive nanocarriers (BNPs) through the oxidation of the hydroxyl-rich HPG shell. Under mild sodium periodate conditions, adjacent vicinal diols on the HPG corona are selectively oxidized to form aldehyde groups (Figure 4D). These reactive aldehydes can rapidly and selectively form Schiff-base linkages with the ε-amino groups of skin proteins, particularly lysine residues, creating stable, yet reversible, covalent bonds. This surface modification facilitates secure, noninvasive adhesion of the nanocarriers to the outer skin layer, enhancing retention without damaging underlying tissues.

In vivo experiments conducted on pig skin models confirmed the enhanced retention behavior of these BNPs. After standardized washing procedures, BNP-treated areas retained significantly more nanocarriers compared to their nonadhesive counterparts, underscoring the advantage of the aldehyde-amine bonding strategy (Figure 4E,F). Importantly, this adhesion was not permanent. The BNPs could still be removed via gentle mechanical wiping (Figure 4G,H), offering the potential for controlled and reversible application cycles. Furthermore, imaging and histological analyses revealed that, unlike unmodified NPs, which tend to accumulate within hair follicles, the BNPs were predominantly confined to the skin’s surface (Figure 4I,J). This surface localization minimizes the risk of follicular occlusion, thereby reducing the incidence of irritation or adverse effects such as acneiform eruptions.

In vivo safety and efficacy assessments further reinforced the potential of BNPs as next-generation sunscreen delivery systems. Even at low UV-filter concentrations (< 5%), comparable to or lower than those in commercial sunscreens, BNPs achieved effective photoprotection. Histological sections from untreated skin displayed features of UV damage, including acanthosis and elongation of rete ridges. In contrast, skin treated with BNPs maintained normal epidermal architecture (Figure 4K,L). Notably, conventional sunscreens led to orthokeratosis, a condition indicative of disrupted follicular function and commonly associated with keratosis pilaris. BNP-treated skin, however, showed no such pathology, highlighting its superior non-irritant profile.

Chronic exposure studies further confirmed the excellent biocompatibility of BNPs. Repeated applications over extended durations did not induce visible inflammation, erythema, edema, or other signs of dermal toxicity (Figure 4M). Beyond sunburn prevention, BNPs were also effective in mitigating UV-induced DNA damage. Specifically, they suppressed the formation of cyclobutane pyrimidine dimers (CPDs), which are major UVB-induced DNA lesions implicated in photocarcinogenesis (Figure 4N,O). Interestingly, although both BNPs and commercial sunscreens reduced CPD levels, only the latter significantly increased γH2AX expression—a biomarker for double-strand DNA breaks (DSBs). This suggests that commercial formulations may generate reactive oxygen species (ROS) capable of secondary DNA damage. In contrast, BNPs maintained γH2AX levels near baseline and appeared to contain ROS activity (Figure 4P), possibly through intrinsic scavenging effects or barrier stabilization.

Finally, under acute UVB exposure, BNPs provided strong protective effects, comparable to or exceeding those of traditional sunscreens. No visible erythema or edema was observed in BNP-treated areas, whereas untreated controls exhibited significant inflammatory responses (Figure 4Q). These findings collectively suggest that BNPs represent a biocompatible, noninvasive, and functionally superior alternative to conventional sunscreen systems, with potential applications extending to other topical therapies requiring surface-specific, reversible delivery.

**Figure 5:**
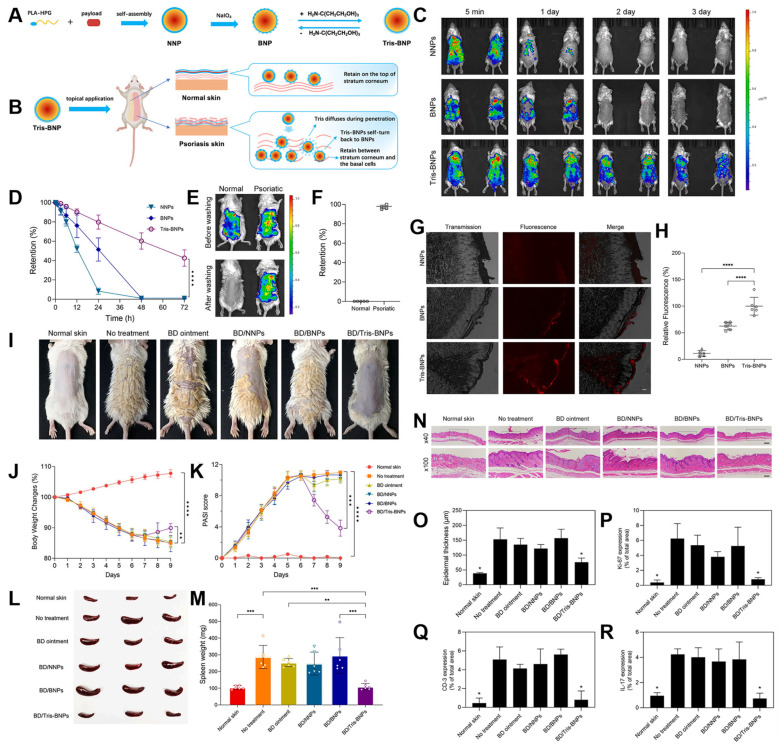
(**A**) The conversion of Tris-BNPs from BNPs and NNPs. (**B**) The Tris-BNPs were applied onto the normal skin and psoriasis skin. (**C**) NNPs, BNPs, and Tris-BNPs encapsulating Cy5 were added on the dorsal skin of IMQ-induced psoriasis model mice, and their skin retention was imaged with Xenogen at different time points. (**D**) The fluorescence was quantified and normalized to the fluorescence intensity at time zero. (**E**) Tris-BNPs encapsulating Cy5 were applied onto the dorsal skin of normal and psoriatic mice. After washing with water and wiping with a towel, their skin retention was imaged with Xenogen. (**F**) The remaining fluorescence after washing and drying on normal and psoriatic skin was quantified and normalized to the fluorescence intensity before washing. (**G**) NNPs, BNPs, and Tris-BNPs at 1 mg/mL were added to psoriatic skin from human volunteers. (**H**) The fluorescence on human skin was quantified and normalized to the average fluorescence of Tris-BNPs. (**I**) Photographs of healthy mice and psoriatic mice treated with various formulations once after 3 days, including commercial betamethasone dipropionate (BD) ointment, BD/NNPs, BD/BNPs, and BD/Tris-BNPs. (**J**) The body weight change during the treatment. (**K**) PASI scores were recorded every day for 9 days until the end of the experiment. (**L**) Representative images of the spleens at day 9. (**M**) Spleen weights at day 9. (**N**) H&E staining of the skin tissue sections from the healthy mice or IMQ-induced psoriasis mice after various treatments, including commercial betamethasone dipropionate (BD) ointment, BD/NNPs, BD/BNPs, and BD/Tris-BNPs. (**O**) Epidermal thicknesses were measured in H&E-stained microphotographs. (**P**) Percentage of Ki-67 compared to the total in different treatment groups. (**Q**) Percentage of CD3 compared to total in different treatment groups. (**R**) Percentage of IL-17 compared to total in different treatment groups. Reproduced with permission from [35]. Copyright 2022 Elsevier. B.V.

Beyond photoprotection, BNPs have also demonstrated promising potential as platforms for topical drug delivery in the management of chronic inflammatory skin diseases, particularly psoriasis. Psoriasis is characterized by aberrant immune activation, excessive keratinocyte proliferation, and impaired barrier function, which together pose significant challenges for effective transdermal drug delivery. In this context, Mai et al. developed a disease-specific nanocarrier system designed for a prospective single-dose therapy targeting psoriatic lesions (Figure 5) [35].

To address the altered barrier properties of psoriatic skin, the authors engineered tris(hydroxymethyl)aminomethane (Tris)-modified BNPs, or Tris-BNPs. These carriers were designed with temporarily reduced adhesiveness, allowing them to bypass the hyperkeratotic stratum corneum and achieve deeper drug penetration. This tunable bioadhesion strategy represents a key innovation for maximizing drug deposition within inflamed dermal layers while minimizing off-target accumulation (Figure 5A,B).

Using an imiquimod-induced mouse model of psoriasis—a well-established in vivo system that closely mimics human psoriatic inflammation—the researchers demonstrated that Tris-BNPs exhibited significantly enhanced retention at the lesion site, maintaining localization for up to 4 days compared to 1–2 days for conventional NPs (Figure 5C,F). Notably, Tris-BNPs also showed preferential binding to psoriatic plaques, suggesting lesion-specific accumulation. Depth profiling revealed that the carriers achieved penetration depths of 128.3 ± 57.1 μm in murine skin and 414.5 ± 100.0 μm in ex vivo human skin (Figure 5G,H), surpassing typical passive diffusion limitations in hyperproliferative epidermis.

When loaded with the potent corticosteroid betamethasone, a first-line treatment for moderate-to-severe psoriasis, Tris-BNPs significantly improved therapeutic outcomes. A single topical application led to marked reductions in Psoriasis Area and Severity Index (PASI) scores (Figure 5I,K), alongside suppression of systemic inflammatory markers such as spleen size and circulating cytokines (Figure 5L,M).

Histological assessments further supported the therapeutic efficacy of this system. Compared to untreated or conventionally treated controls, skin sections from Tris-BNP-treated mice displayed reduced epidermal thickness, diminished parakeratosis, and attenuated inflammatory cell infiltration (Figure 5N,P). Immunohistochemical analysis revealed substantial downregulation of CD3+ T cells, indicative of reduced T-lymphocyte infiltration into lesional skin. Additionally, expression of Ki-67, a marker of keratinocyte proliferation, was decreased, suggesting normalization of epidermal turnover. Levels of interleukin 17 (IL-17)—a proinflammatory cytokine central to the pathogenesis of psoriasis—were also markedly reduced (Figure 5Q,R), demonstrating broad immunomodulatory effects.

### 4.2. Wound Dressing

Burn wounds, particularly those complicated by methicillin-resistant Staphylococcus aureus (MRSA) infections, present a formidable clinical challenge due to their abundant wound exudate, high susceptibility to biofilm formation, and protracted healing processes. These factors not only impair tissue regeneration but also significantly reduce the efficacy of systemic antibiotics, especially in the presence of biofilms that act as physical and biochemical barriers to antimicrobial agents. The development of therapeutic platforms capable of simultaneously addressing infection control and wound tissue repair is therefore of critical importance.

**Figure 6:**
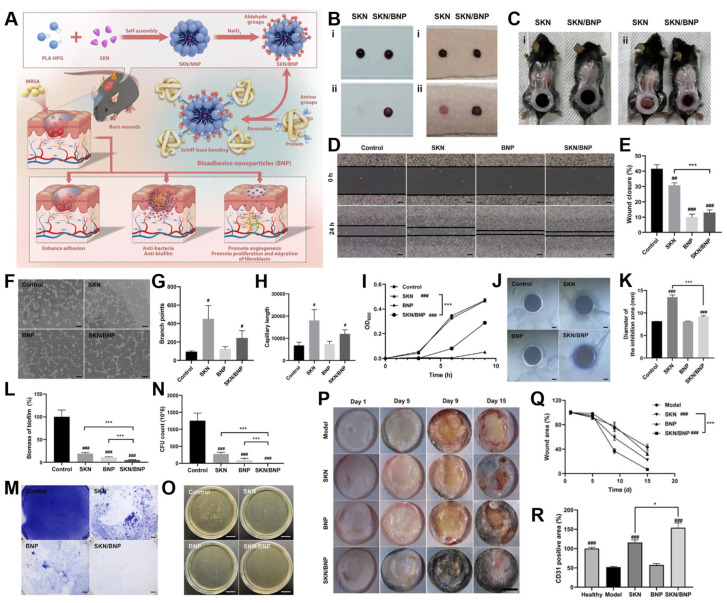
(**A**) Diagram illustrating the synergistic effect of BNP and SKN assembled as SKN/BNP in promoting burn wound healing. (**B**,**C**) Evaluation of SKN/BNP adhesion. Retention of SKN and SKN/BNP on (**B**) polylysine-coated slides, pig skin, and (**C**) burn wound in mice. SKN and SKN/BNP at 1.5 mg/mL were applied individually, followed by incubation at 32 °C for 0.5 h (for polylysine-coated slides and pig skin) or 2 h (for burn wound). After washing with PBS thoroughly, the retention was imaged. (**D**) Effects of SKN, BNP, and SKN/BNP on L929 migration, and (**E**) the wound closure distance was quantified. (**F**) Effects of SKN, BNP, and SKN/BNP on promoting angiogenesis of EA.hy926, and (**G**) branch points and (**H**) capillary length were measured using ImageJ. The L929 and EA.hy926 cells were treated with 1 μM SKN, 160 μg/mL BNP, and 20 μM SKN/BNP. (I) Bacterial growth curves of MRSA. (**J**,**K**) The effect of 20 μM SKN, 3.2 mg/mL BNP, and 400 μM SKN/BNP on the inhibition zone of MRSA. (**L**,**M**) The biomass of residual MRSA biofilm. The biofilm was stained with crystal violet. (**N**) The number of live bacteria in remaining MRSA biofilm. (**O**) Representative photographs of MRSA colonies. (**P**) Photographs of MRSA-infected burn wounds after different treatments. (**Q**) Quantification of unhealed wound area. (**R**) Quantification of CD31 expression in burn wounds. # *p* < 0.05; ## *p* < 0.01; ### *p* < 0.001, compared with the control group. * *p* < 0.05; *** *p* < 0.001. Reproduced with permission from [38]. Copyright 2023 Elsevier B.V. All rights reserved.

In a recent study, bioadhesive nanocarriers based on PLA-HPG copolymers (BNPs) were utilized to encapsulate shikonin (SKN)—a hydrophobic phytochemical extracted from Lithospermum erythrorhizon, known for its antibacterial, anti-inflammatory, anti-biofilm, and wound-healing-promoting properties [38]. The resultant formulation, SKN-loaded BNPs (SKN/BNPs), aimed to combine sustained antimicrobial efficacy with localized regenerative support for treating MRSA-infected burn wounds (Figure 6A).

Adhesion experiments using porcine skin and polylysine-coated surfaces revealed that SKN/BNPs exhibited markedly enhanced surface retention compared to free SKN. This was attributed to the aldehyde-modified HPG shell, which forms stable Schiff-base bonds with amine-rich proteins in the wound environment. The bioadhesive capability of SKN/BNPs was validated in a simulated exudative wound model, where the carriers maintained robust adhesion even after extensive phosphate-buffered saline (PBS) washing, confirming their suitability for sustained topical application (Figure 6B,C).

Beyond adhesion, the system facilitated wound healing through multifactorial mechanisms. Both free SKN and SKN/BNPs were observed to stimulate fibroblast migration, an essential process for granulation tissue formation and wound contraction (Figure 6D,E). The formulation also preserved angiogenic capacity, as demonstrated by the formation of capillary-like networks in endothelial cell assays. While the SKN/BNPs exhibited slightly reduced tube formation efficiency compared to free SKN (Figure 6F,H), this was likely a result of the controlled drug release profile that minimized peak exposure while sustaining therapeutic levels over time.

In terms of antimicrobial efficacy, SKN at 20 μM completely inhibited the growth of MRSA over a 9 h period, consistent with its known mechanisms involving membrane disruption and oxidative stress induction. In contrast, SKN/BNPs showed partial bacterial growth suppression, attributable to the gradual, controlled release of SKN (Figure 6I,K). Although the immediate bactericidal effect was moderated, the sustained release profile may offer long-term advantages in reducing bacterial regrowth and resistance development.

The most striking result of this system was its anti-biofilm performance, a major limitation of most conventional topical antimicrobials. Crystal violet staining revealed that while free SKN alone could reduce biofilm biomass through direct bactericidal action, BNPs alone unexpectedly compromised bacterial structural integrity, possibly by physically interacting with and disrupting extracellular polymeric substances, thereby rendering the biofilm more susceptible to mechanical clearance (Figure 6L,M). When combined, the SKN/BNP system demonstrated a synergistic effect, achieving superior biofilm penetration and bacterial eradication compared to either component alone (Figure 6N,O).

These in vitro outcomes were further corroborated by in vivo experiments in an MRSA-infected burn model. SKN/BNP treatment led to a marked reduction in visible wound biofilm accumulation, significant acceleration of wound closure, and enhanced neovascularization, as evidenced by increased CD31+ microvessel density (Figure 6P,R). This holistic therapeutic effect—encompassing bacterial suppression, biofilm disruption, and tissue regeneration—underscores the potential of SKN/BNPs as a multifunctional, bioadhesive nanoplatform for the localized treatment of infected burn wounds, particularly those resistant to conventional antibiotics.

In another innovative study, Liang et al. explored the multifunctional therapeutic potential of PLA-HPG-based bioadhesive nanocomposite hydrogels (NC gels) to tackle methicillin-resistant Staphylococcus aureus (MRSA) biofilms through a synergistic triple-therapy strategy [39]. The system was constructed by integrating bioadhesive PLA-HPG nanocarriers (BNPs) with carboxymethyl chitosan (CS) to form a hydrogel matrix capable of delivering three distinct therapeutic agents: S-nitrosothiol (SNO) as a nitric oxide (NO) donor; amylase (Am) for enzymatic degradation of biofilm matrix; and cefepime (Cef), a broad-spectrum fourth-generation cephalosporin antibiotic.

**Figure 7:**
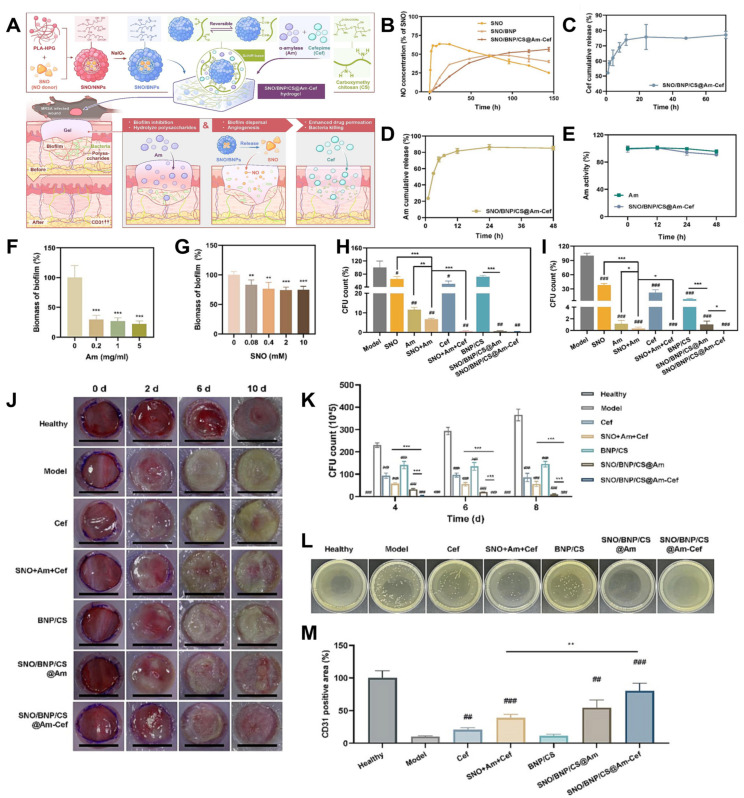
(**A**) Schematic illustration of the preparation and enhanced efficacy of co-delivering NO, Am, and Cef by the BNP/CS NC gel for chronic wounds with biofilm infections. (**B**) Release profile of NO from SNO, SNO/BNPs, and SNO/BNP/CS@Am-Cef NC gel. (**C**,**D**) Release profile of Cef (**C**) and Am (**D**) from SNO/BNP/CS@Am-Cef NC gel. (**E**) Enzymatic activity of Am in SNO/BNP/CS@Am-Cef NC gel. (**F**,**G**) MSSA biofilm dispersal with Am and (**F**,**G**) SNO at a range of concentrations. (**H**,**I**) The number of live bacteria in MSSA biofilm (**H**). MRSA biofilm (**I**) after treatments with PBS, Am, SNO, SNO+Am, Cef, SNO+Am+Cef, blank BNP/CS, SNO/BNP/CS@Am, or SNO/BNP/CS@Am-Cef NC gel. All treatment groups contained equal doses of Am, SNO, and Cef. (**J**) Representative images of wound change after treatments in mice infected with MRSA. (**K**) Quantification of viable bacteria inside biofilms at 4, 6, and 8 d post-modeling. (**L**) Representative photographs of MRSA colonies from biofilms at 6 d post-modeling. (**M**) Quantitative analysis of CD31 in wound tissues on day 9. # *p* < 0.05; ## *p* < 0.01; ### *p* < 0.001, compared with the control group. * *p* < 0.05; *** *p* < 0.001. Reproduced with permission from [39]. Copyright 2023 The Authors.

The design leveraged the porous architecture of the NC gel, which allowed for efficient loading of the hydrophilic agents Am and Cef within the CS hydrogel network, while the hydrophobic SNO was encapsulated within the BNPs. This dual compartmentalization enabled tailored release profiles for each therapeutic component (Figure 7A). Specifically, BNP encapsulation of SNO significantly prolonged NO release, extending its therapeutic window from the typical short-lived burst (a few hours) to a sustained release over three days, a critical feature for disrupting biofilms and promoting tissue regeneration (Figure 7B).

Further release kinetics studies substantiated the effectiveness of this delivery strategy. Free SNO exhibited a rapid burst release, compromising its sustained antimicrobial potential. In contrast, SNO/BNPs and the full composite SNO/BNP/CS@Am-Cef formulation both achieved controlled, extended NO release, demonstrating the importance of the BNP-CS composite matrix in modulating diffusivity and maintaining NO bioavailability over time (Figure 7B). Likewise, Cefepime release was significantly delayed compared to free-drug formulations (Figure 7C), enabling prolonged antibacterial action. Meanwhile, Amylase exhibited a steady, sustained release profile, achieving approximately 62% cumulative release over 24 h, which is notably improved compared to typical hydrogel systems that often suffer from undesirable initial burst release. This was attributed to Schiff-base formation between the aldehyde-functionalized CS matrix and the amine groups on Am, which facilitated reversible covalent immobilization and prolonged retention (Figure 7D).

Crucially, the formulation was able to preserve the enzymatic activity of Am for up to 48 h post-encapsulation, ensuring that the biofilm-degrading function was retained throughout the therapeutic period (Figure 7E). This is essential for overcoming the extracellular polymeric substance (EPS) barrier, a key structural component of biofilms that impedes antibiotic penetration.

The anti-biofilm efficacy of the NC gel was validated through a dual-mechanism approach. First, Am directly degraded the EPS matrix, compromising the biofilm’s physical integrity. Second, NO released from SNO acted as a quorum-sensing inhibitor and dispersal agent, facilitating detachment of bacteria from the biofilm and enhancing susceptibility to antibiotics. While standalone application of Am or SNO demonstrated only modest reductions in biofilm biomass (Figure 7F,G), their combined application within the NC gel dramatically enhanced efficacy. The composite SNO/BNP/CS@Am-Cef system reduced the viability of MSSA and MRSA biofilm-embedded bacteria to below 0.1%, far surpassing the performance of free-drug combinations (Figure 7H,I).

These in vitro findings translated into compelling in vivo performance. In a murine MRSA-infected wound model, animals treated with free SNO + Am + Cef showed only marginal reductions in bacterial burden. In stark contrast, those treated with the NC gel exhibited near-complete biofilm eradication by day 10 post-treatment (Figure 7J,L), demonstrating the value of sustained, localized, and multi-modal intervention.

In addition to its anti-infective capabilities, the NC gel also promoted tissue regeneration. Immunohistochemical staining for CD31, a marker for endothelial cells, revealed a 2.3-fold increase in microvessel density in NC gel-treated wounds compared to controls (Figure 7M), highlighting the pro-angiogenic effects of sustained NO delivery. This enhancement in neovascularization is crucial for nutrient delivery, waste removal, and granulation tissue formation, thereby supporting the comprehensive repair of infected wounds.

### 4.3. Tumor-Targeted Drug Delivery

**Figure 8:**
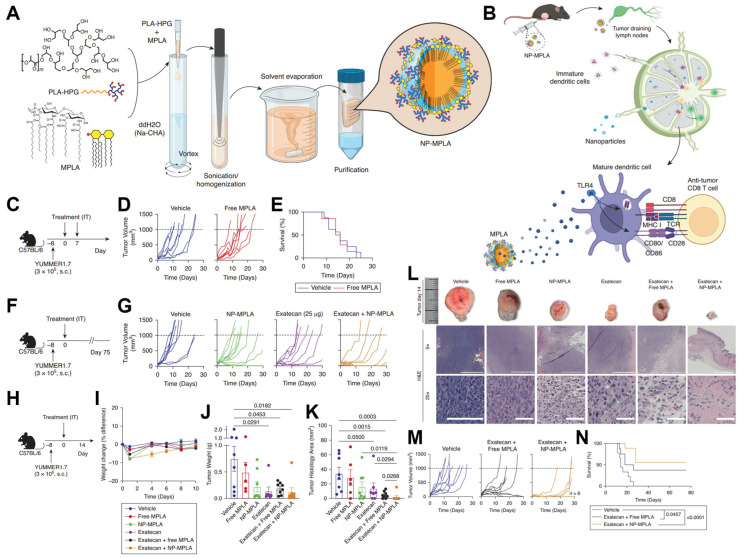
(**A**) Scheme of preparation of nonadhesive biodegradable NPs with MPLA encapsulation. (**B**) Schematic showing the potential distribution of NP-encapsulated MPLA in TDLNs and interaction with DCs. (**C**) Timeline for D and E. Tumor-bearing mice were treated with 2 once-weekly IT injections of vehicle or free MPLA. (**D**) Individual tumor growth curves and (**E**) corresponding survival curve. (**F**) Timeline for G. Tumor-bearing mice were treated with a single IT injection of vehicle, NP-encapsulated MPLA, Exatecan, or Exatecan plus NP-encapsulated MPLA. (**G**) Individual tumor growth curves. (**H**) Timeline for I to N. Tumor-bearing mice were treated with a single IT injection of vehicle, free MPLA, NP-encapsulated MPLA, Exatecan, free MPLA plus Exatecan, or NP-encapsulated MPLA plus Exatecan and harvested 14 days later. (**I**) Mouse weight. (**J**) Harvested tumor weight. (**K**) Histologic tumor area. (**L**) Representative tumors and histology. (**M**) Tumor growth curves and (**N**) survival curves from a second cohort treated as described earlier with vehicle, free MPLA plus Exatecan, or NP-encapsulated MPLA plus Exatecan. Reproduced with permission from [40]. Copyright 2024 The Authors.

The design and construction of tumor-targeted nanocarriers have attracted more interest in recent years because of the clinical demand for targeted and sustained drug delivery with reduced systemic toxicity. In a study conducted by Chang et al., PLA-HPG NPs were investigated for the treatment of melanoma [40]. To address the delivery challenges and limited efficacy of the immunomodulator Monophosphoryl lipid A (MPLA), it was encapsulated within PLA-HPG NPs (Figure 8A). The central hypothesis posited that co-delivering this NP-encapsulated MPLA alongside a chemotherapeutic agent would enhance antitumor immunity by remodeling the tumor microenvironment and activating tumor-draining lymph nodes (TDLNs) (Figure 8A).

Initial experiments confirmed the necessity for an enhanced strategy, as modulating the TDLNs (Figure 8B) and treating tumors with free MPLA alone were insufficient to slow melanoma progression (Figure 8C,E). The study then shifted to a combination therapy involving the chemotherapeutic agent exatecan, which significantly inhibited tumor growth when paired with NP-encapsulated MPLA, far surpassing the effects of either agent alone (Figure 8F,G). This combination was well tolerated, with mice quickly recovering from minor, temporary weight loss (Figure 8H–J). The combination therapy resulted in significantly decreased tumor weight (Figure 8J) and complete tumor eradication in 87.5% of mice, a stark contrast to the 25% clearance observed with free MPLA plus chemotherapy (Figure 8K,L). An enhanced regimen that included an additional immunomodulator injection (Figure 8M) proved to be the most effective, achieving complete tumor resolution in 56% of treated mice and demonstrating significant potential for improving clinical outcomes (Figure 8N).

PLA-HPG nanocarriers possess distinctive structural and surface chemical features that enable them to overcome biological barriers such as the blood–brain barrier (BBB) and the tumor microenvironment (TME). HPG shell forms a dense hydration layer, which provides a ’stealth’ property to evade immune surveillance and prolong the circulation time [22,32]. Additionally, when the HPG shell is functionalized with aldehyde groups to form BNPs, these particles can form covalent Schiff-base bonds with amine-rich components on cell membranes and extracellular matrix proteins, promoting selective adhesion and retention within targeted tissues [41,42]. This bioadhesive capability is critical for enhancing tissue penetration, local accumulation, and sustained therapeutic effect, particularly in dense tumor matrices and across restrictive barriers like the BBB.

**Figure 9:**
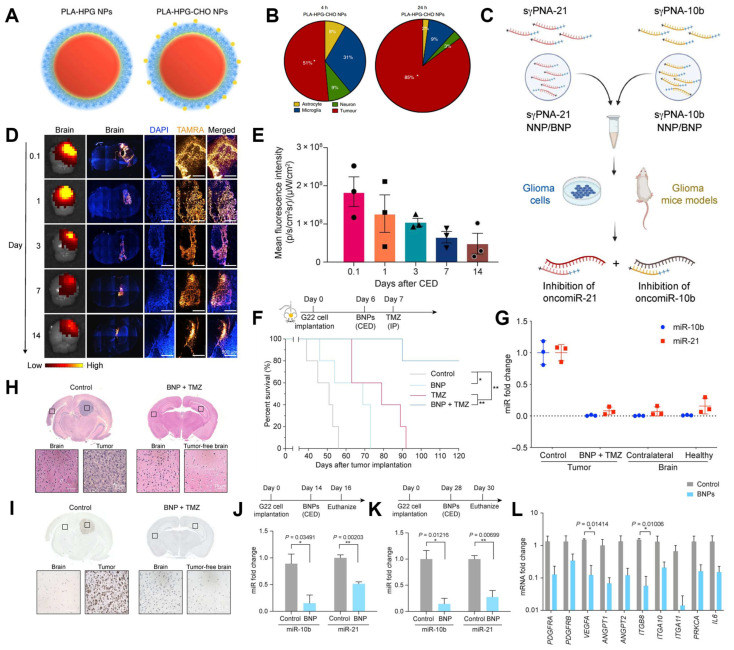
(**A**) Schematic of PLA-based NPs with different surface coatings. (**B**) Cellular tropism of PLA-HPG-CHO NPs 4 and 24 h after CED in the tumor-bearing brain. Reproduced with permission from [41]. Copyright 2017 The Authors. (**C**) Graphical representation of sγPNA encapsulation in NPs (NNP or BNP) and treatment strategy for the simultaneous inhibition of oncomiRs 21 and 10b. (**D**) Biodistribution of BNPs in U87 orthotopic mice model of GBM after CED. (**D**) From left to right, Columns 1 and 2: IVIS and microscopic images of mice brain at days 0.1 (3 h), 1, 3, 7, and 14 after CED of TAMRA-labeled sγPNA BNP into the tumor. CED was performed 10 days after tumor inoculation. Columns 3 to 5: Microscopic images showing the injection site of the brain sections at different time points with a higher magnification (1 × objective). Nucleus is shown in blue (DAPI), and sγPNA (TAMRA) is shown in yellow. (**E**) Mean fluorescence intensity of TAMRA-sγPNAs in the brain IVIS images of (**D**) at different time points. (**F**) Survival of mice-bearing patient (G22)–derived intracranial gliomas after treatment with BNPs containing sγPNA, TMZ, and the combination treatment of BNP with TMZ. (**G**) The levels of miR-21 and miR-10b in gliomas of control and BNP + TMZ-treated mice at the end of the survival study. (**H**) Histology of H&E-stained control and BNP + TMZ-treated mice brain at the end of the survival study. Control mouse brain was harvested on day 39, and sγPNA/BNP + TMZ-treated mouse brain was harvested on day 120. (**I**) Ki67 staining of control and BNP + TMZ-treated mice brain at the end of the survival study. Control mouse brain was harvested on day 39, and sγPNA/BNP + TMZ-treated mouse brain was harvested on day 120. (**J**) The expression levels of miR-10b and miR-21 in mice gliomas 48 h after CED of NPs on day 14 and (**K**) day 28 after tumor implantation. (**L**) The levels of downstream genes PDGFRA, PDGFRB, VEGFA, ITGB8, ITGA10, ITGA11, ANGPT2, ANGPT1, PRKCA, and IL6 in mice gliomas 48 h after CED of NPs on day 28. sγPNA/BNPs are a physical mixture of sγPNA-21 BNP and sγPNA-10b BNP. NNP indicates PLA-HPG NP, and BNP indicates PLA-HPG CHO NP. * *p* < 0.05; *** *p* < 0.001. Reproduced with permission from [42]. Copyright 2023 The American Association for the Advancement of Science.

For example, Song et al. discovered that BNPs, created by modifying their surfaces with aldehydes (Figure 9A), exhibit markedly increased selective uptake by tumor cells (Figure 9B) [41]. In the work of Wang et al., the BNPs were used to encapsulate peptide nucleic acids (PNAs) for the simultaneous knockdown of the oncogenic microRNAs (oncomiRs) 10b and 21 (Figure 9C) [42]. Following direct infusion into intracranial tumors via convection-enhanced delivery (CED) (Figure 9D), the BNPs demonstrated sustained cargo retention within the tumor for up to two weeks (Figure 9E). The therapeutic efficacy of this approach was evaluated in a patient-derived glioblastoma (GBM) model; combining the PNA-loaded BNPs with standard chemotherapy, temozolomide (TMZ), significantly prolonged survival: 80% of the animals in this group survived to the 120-day endpoint (Figure 9F). This represented a significant improvement over the modest survival extension provided by either PNA/BNP or TMZ alone (Figure 9G). Histological analysis revealed that the brains of the surviving animals were tumor free (Figure 9H,I). Furthermore, the therapy achieved highly efficient knockdown of the target miR-10b (99%) and miR-21 (92%) (Figure 9J,K), along with substantial downregulation of key downstream oncogenes, thereby supporting the mechanism of action and survival improvement in the orthotopic brain tumor model (Figure 9L).

**Figure 10:**
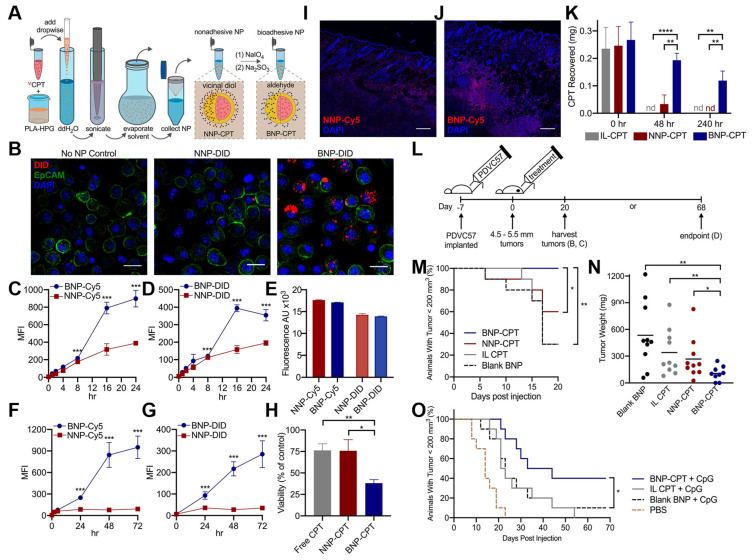
(**A**) Synthesis and bioadhesion of CPT-loaded PLA-HPG NNPs and BNPs. (**B**) Confocal microscopy of PDV SCC cells following 24 h incubation with DiD dye-loaded NNPs and BNPs demonstrate superior affinity of BNPs to tumor cells. (**C**–**E**) Assessment of cellular association over time via flow cytometric analysis of PDV cultured under serum-free conditions with Cy5-conjugated NPs (**C**) and DiD-loaded NPs (**D**) demonstrates significantly improved association of BNP in both cases, with their advantage especially notable at later time points (16 to 24 h). Baseline fluorescence (**E**) of both dye-conjugated (Cy5) and -loaded (DiD) NNP and BNP before incubation is similar. (**F**,**G**) Cellular association of Cy5-NPs (**F**) and DiD-NPs (**G**) with extended incubation in serum-containing media. Even with prolonged exposure, BNPs associate with tumor cells more strongly than NNPs. (**H**) PDVC57 was incubated with free CPT, NNP-CPT, or BNP-CPT for 1 h. The CPT solutions were then aspirated and replaced with fresh media for 48 h, when viability was assessed. BNP-CPT increased tumor cell cytotoxicity compared to all other groups. (**I**,**J**) Confocal microscopy of PDVC57 tumors and overlying skin 72 h after injection of NNP-Cy5 (**I**) or BNP-Cy5 (**J**). BNP-Cy5 exhibited greater intra-tumoral retention when compared to NNP-Cy5 (fluorescence integrated density/tissue area: NNP, 3541.88; BNP, 10402.26). (**K**) CPT recovered from tumors after treatment with intralipid-CPT (IL-CPT), NNP-CPT, or BNP-CPT. At 48 and 240 h following treatment, a greater percentage of the injected CPT was recovered from tumors treated with BNP-CPT when compared to free CPT and NNP-CPT. (**L**) Experiment timeline. Tumors injected with BNP-CPT showed significantly delayed tumor growth (**M**) and reduced tumor weight at harvest (**N**) when compared to groups treated with either blank BNPs, IL-CPT, or NNP-CPT. (**O**) Combination therapy with BNP-CPT plus the TLR9 agonist, CpG, significantly delayed tumor growth. Reproduced with permission from [43]. Copyright 2021 The Authors.

Hu et al. found that BNPs developed to encapsulate the chemotherapy drug CPT enhanced its delivery and tumor elimination compared to NNP or free drug controls (Figure 10A) [43]. In vitro studies demonstrated that BNPs had significantly greater adhesion and association with tumor cells than NNPs, as observed through microscopy (Figure 10B) and quantified by flow cytometry in both serum-free (Figure 10C–E) and serum-containing media over extended periods (Figure 10F,G). This superior binding led to significantly greater tumor cell toxicity from BNP-CPT in a simulated tumor matrix (Figure 10H). In an in vivo murine cutaneous squamous cell carcinoma (SCC) model, BNPs showed sustained distribution and greater retention within the tumor (Figure 10I,J). Quantitatively, 45% of the CPT from BNPs remained in the tumor for up to 10 days, while most of the CPT from control groups was undetectable, showing improved drug retention (Figure 10K). This translated into a direct therapeutic benefit, as mice treated with BNP-CPT exhibited considerably delayed tumor development and decreased tumor weight (Figure 10L–N). Finally, the addition of the immunostimulatory agent CpG to the BNP-CPT therapy resulted in even greater tumor growth delay and complete tumor resolution in 20% of the mice, presenting a viable and practical nonsurgical approach for skin cancer treatment (Figure 10O).

## 5. Conclusions and Perspective

In this review, we describe the synthesis methods, physicochemical properties, and biomedical applications of PLA-HPG-based biodegradable nanocarriers. These amphiphilic NPs feature a hydrophobic PLA core and a hydrophilic HPG corona, fulfilling the promise of biodegradability, biocompatibility, and versatile surface properties. The hyperbranched structure of HPG facilitates dense hydration and imparts antifouling properties, while PLA ensures sustained release and structural integrity. PLA-HPG nanocarriers are distinct from other established systems, such as PEG-PLA or PLA-PCL, due to the unique combination of high-density hydroxyl groups on the HPG corona.

Despite these promising attributes, several key challenges still hinder the clinical translation of PLA-HPG-based nanocarriers. First, the drug loading capacity—especially for hydrophilic or high-molecular-weight therapeutics—remains suboptimal, limiting their application scope. Second, uncertainties surrounding long-term biocompatibility and potential immunogenicity must be carefully addressed, as even minimal immune activation could compromise repeated dosing regimens. Third, the scalable and reproducible synthesis of well-defined PLA-HPG copolymers remains technically challenging, especially for maintaining narrow polydispersity and batch-to-batch consistency. Fourth, regulatory hurdles for complex hybrid nanocarriers—particularly those incorporating multifunctional or responsive components—are still poorly defined, slowing clinical translation.

In addition, material-intrinsic properties may further complicate in vivo performance. For example, the high hydrophilicity of HPG, while beneficial for antifouling and colloidal stability, can lead to increased water absorption, which in turn accelerates the hydrolysis of ester bonds in PLA blocks. This premature degradation may compromise drug retention and lead to uncontrolled release profiles under physiological conditions. To address this, careful tuning of the PLA/HPG ratio and the molecular weight of PLA segments is essential to modulate degradation kinetics. Optimizing the hydrophilic content of HPG can help balance water uptake and maintain sustained drug release, thereby preventing rapid hydrolysis in vivo.

Moreover, recent research suggests PLA-based systems can be engineered with advanced functionalities, including co-delivery capacity, scaffold integration, and responsiveness to external stimuli such as pH, redox conditions, enzymes, ultrasound, and magnetic fields [44]. For example, Malekmohammadi et al. designed PLA nanofibers embedded with growth factors and ultrasound-responsive modules, achieving synergistic osteogenesis outcomes in tissue regeneration applications [45]. Building on this concept, integrating multifunctional and stimuli-responsive elements into PLA-HPG nanocarriers may further expand their biomedical utility by enabling spatiotemporally controlled drug release tailored to specific pathophysiological environments [44,45]. This strategy enhances functionalization flexibility and biocompatibility while significantly reducing the immunogenic response and mitigating the accelerated blood clearance effect—a known limitation of PEGylated systems. As a result, PLA-HPG nanocarriers can offer prolonged circulation times and improved immune evasion, making them highly suitable for both systemic and localized therapeutic delivery in precision medicine applications.

## Figures and Tables

**Table 1:** Comparison of synthesis methods for HPG and PLA-HPG copolymers.

Method	Control Over MW	Polydispersity	Biocompatibility of Reagents	Scalability
Anionic Polymerization	Excellent	~1.2	Moderate, requires basic initiators	Moderate, limited by initiator toxicity
Cationic Polymerization	Moderate	~1.3–1.6	Variable, dependent on catalysts	Not easily scalable due to catalyst
ROMBP	Excellent	<1.3	High, uses non-toxic initiators	High, requires precise control of conditions
Ascorbic Acid Initiated	Moderate	~1.5	Excellent, biocompatible activator	High, no toxic solvents or reagents
Amino Coupling	Moderate	~1.5	Moderate, carbodiimide and amine activation	Moderate, efficient but sensitive to excess unreacted amines
Direct Esterification	Moderate	~1.3	High, uses simple coupling reagents	High, straightforward and efficient

**Table 2:** Summary of patents and applications of PLA-HPG nanocarriers in biomedical fields.

Patent Number	Application Area	Key Features	Delivery Form
CN117679393	Hypertrophic scar treatment	Vitepofen-loaded, local-adhesion, controlled-release	PLA-HPG sustained-release particles
WO2022165403/US20240423972	Cancer therapy	CPT delivery with immune adjuvants	Bioadhesive PLA-HPG NPs
CN115531349	Bacterial conjunctivitis	PEG network system enhances ocular retention	PPHNP-AL-PEG adhesive NP system
CN115737598	Brain disease treatment	Intranasal BNP-PAMAM cluster targets CNS	Excellent, biocompatible activator
WO2023060716/CN113975249	Psoriasis	Tris neutralization reversibly masks adhesiveness	Al-PHNPs-PAMAM nano cluster
CN116327965	Skin drug delivery	Schiff-base hydrogel network enhances dual drug	Variable, dependent on catalysts
CN113876716	Gastrointestinal diseases	GI adhesion prolongs local effect	High, uses non-toxic initiators
CN116832011	Scald wound	SKN-loaded BNP prolongs local effect	Excellent, biocompatible activator
WO2023220067/US20230355801	Cancer immunotherapy	MPLA-loaded BNPs improve dendritic cell response and lymphatic targeting	Excellent, biocompatible activator
